# Eucalyptus ash alters secondary protein conformation of human grey hair and facilitates anthocyanin dyeing

**DOI:** 10.1371/journal.pone.0199696

**Published:** 2018-07-02

**Authors:** Aroonsri Priprem, Yao-Chang Lee, Wanwisa Limphirat, Suppachai Tiyaworanant, Kedsarin Saodaeng, Jiranan Chotitumnavee, Nuttanunth Kowtragoon

**Affiliations:** 1 Department of Pharmaceutical Technology, Faculty of Pharmaceutical Science, Khon Kaen University, Muang, Khon Kaen, Thailand; 2 National Synchrotron Radiation Research Center, Hsinchu City, Taiwan, Republic of China; 3 National Synchrotron Research Institute of Thailand, Muang, Nakornratchasima, Thailand; 4 Department of Pharmacognosy and Toxicology, Faculty of Pharmaceutical Science, Khon Kaen University, Muang, Khon Kaen, Thailand; 5 Postgraduate program, Master degree in Pharmaceutical Chemistry and Natural Products, Graduate School, Khon Kaen University, Muang, Khon Kaen, Thailand; 6 Undergraduate program in Doctor of Pharmacy, Faculty of Pharmaceutical Science, Khon Kaen University, Muang, Khon Kaen, Thailand; Oregon State University, UNITED STATES

## Abstract

Wood ashes infused with water have been traditionally used as hair cosmetics, but little or no research has examined the effects of ash on human hair. This study investigated the effect of eucalyptus ash on the structure and morphology of excised human grey hair and its potential use as a pretreatment in natural hair dyeing using anthocyanins extracted from purple cops of *Zea mays*. Tensile characteristics and surface morphology of ash-pretreated hair was monitored by texture analysis, scanning electron microscopy and atomic force microscopy. The biochemical characteristics of ash-treated hair were analyzed by synchrotron radiation-FTIR and sulfur *K*-edge X-ray absorption near edge. Dyeing with anthocyanins was analyzed by Lab color scale and adsorption of anthocyanins. Ash-treated hair was elastically and plastically deformed with microscopic alterations to the ridges of the cuticle cells, similar to ammonia-treated hair. The ash extract significantly changed the relative proportion of alpha-helices in the cuticle and cortex layers (p < 0.05), but did not affect the interaction of S-bonds with neighboring atoms (p > 0.05). Ash-treated hair showed significantly enhanced adsorption of anthocyanins (p < 0.05) which changed the color of the grey hair. The alteration of secondary proteins in the cuticle and cortex layers of the grey hair by ash extract pre-treatment, enhanced anthocyanin adsorption. The eucalyptus ash could potentially be useful as a natural hair dyeing pre-treatment.

## Introduction

Wood ash, the product of wood combustion [1], has been traditionally used in several cultures for health [2–5] and cosmetics [4]. Ash obtained from combustion of plants can shift the pH of water into alkaline [5–7] with various inorganic compounds, i.e. potassium (K), calcium (Ca), magnesium (Mg), manganese (Mn), zinc (Zn), iron (Fe), sulfur (S), and phosphorus (P), the proportions of which depend on the processing and type of wood [7–9]. Ash generated from the underutilized industrial waste bark of eucalyptus (*Eucalyptus spp*.) represents one type of wood ash. We studied the effect of eucalyptus ash as a natural alkaline hair pretreatment prior to dyeing with a natural hair dye. Alkalinizing agents such as ammonia and ethanolamine are commonly used as hair pre-treatments to loosen hair scales and remove the natural cuticle lipids, which enables penetration of dyes across the cuticle and into the cortex of the hair shaft [10]. However, strong alkalis and hydrogen peroxide can also oxidize cysteine, resulting in irreversible damage to the structure of the hair [11,12].

Hair strength is affected by various factors, including water sorption [13,14] and the depletion of melanin granules in the cortex of grey hair [15]. The elongation and breakage responses of a hair fiber to increasing tensile forces are used to measure hair strength [13] and chemical changes to hair fibers can be determined by sensitive and precise synchrotron FTIR microspectroscopy [16–19] and X-ray absorption spectroscopy (XAS) [20–22].

Anthocyanins, water-soluble pigments that are extractable from plants [23], are abundant in cobs of purple corn (*Zea mays*) [24–26]. Traditionally, anthocyanin-rich plants have been used as food and in cosmetic dyes or pigments, particularly as a natural hair dye [27]. A natural hair dye prepared from blackcurrant extract showed adsorption of anthocyanin by light blonde human hair [28].

Although wood ash has been traditionally used as a hair treatment, it is not known if wood ash can provide sufficient alkalinity to disrupt the hair cuticle and enhance permeation or adsorption of semi-permanent hair dyes. Similarly, there is no previous study that elaborates the effect of wood ash treatment on the physicochemical characteristics of human hair. In this study, eucalyptus ash and purple waxy corn cobs (*Zea mays* L. var. *Ceratina* Kulesh), as the source of anthocyanin-rich extract [23–25], were selected due to their availability from local agricultural industries that can control several factors potentially affecting the quality of the raw materials, including genetics, planting, processing, and handling procedure during storage and transportation. To proof the physicochemical effects of wood ash on grey human hair and its efficacy as a pre-treatment for a natural hair dye, eucalyptus ash and cobs of *Z*. *mays* were investigated.

## Materials and methods

Ash from the bark of a *Eucalyptus camaldulensis* × *Eucalyptus urophylla* hybrid (H4) was gifted from the Phoenix Pulp and Paper PCL (Khon Kaen, Thailand). Cobs of purple waxy corn (*Zea mays* L. var. *Ceratina* Kulesh var KKU-WX111031) were from Plant Breeding Research Center for Sustainable Agriculture, Faculty of Agriculture, Khon Kaen University. Sodium hydroxide (Merck, Germany), potassium hydroxide (Carlo Erba, France), ammonia solution (NH₃(aq)), Merck, Germany), polyethylene glycol 40 castor oil (Sigma, U.S.A.) and other chemicals were used as received.

UV-visible spectrophotometer (Shimadsu 1240, Japan), microplate reader (Sunrise, Switzerland), light microscope (Olympus IX70 Axio Cam, Germany), scanning electron microscope (SEM, Hitachi S-3000N, Japan), atomic force microscope (AFM, XE120 Park System, Korea), texture analyser (TA.XT plus, Stable Microsystem, U.K.), spectrophotometer colorimeter (HunterLab ColorQuest^®^XE, U.S.A.), sonicator (Ultrasonic Steri-cleaner UC-80, Sturdy, Taiwan) and centrifuge (Kubota 6200, Japan) were used.

### Ash extract

Ash samples collected from industrial waste eucalyptus bark were analyzed by atomic absorption for inorganic compositions by Central Lab Co. Ltd. (Khon Kaen, Thailand) in accordance to AOAC methods. One gram of the ash samples was soaked in 5 g of deionized water at 25 ± 2°C and filtered to obtain ash extract.

### Hair samples

This protocol was submitted to the Institutional Ethical Committee (exemption protocol no. HE591251) prior to study. All samples were grey hair anonymously collected according to the inclusion criteria, i.e grey in color, about 5 cm long cut and 10–30 cm from the scalp. Hair that was waved, straightened or dyed or with fiber diameters of < 60 μm (Digitronic Caliper 110-DBL, Moore and Wright, U.K.) were excluded. Hair samples from five Asian subjects having hair-cut in a local salon were collected by the researchers who de-identified and sorted using a pair of tweezers by one researcher, cleaned by soaking in sodium lauryl sulfate solution (1% w/v) for two min and then gently rinsed in distilled water to remove sebum and particulate matter. All hair samples were placed on clean sheets of paper and stored in tightly-closed storage chambers with a relative humidity (RH) of 55 ± 3%, monitored using a hydrometer. Ash-treated hair was freshly-prepared by soaking grey hair in ash extract (pH 12) for 1 h, rinsed with water (pH 6.8) and blotted dry.

### Preparation of dye solution

Corn cobs were dried, ground and sieved (0.02 mm^2^ pore size) into a fine powder. Six grams of powder were extracted in 100 ml of deionized water at 80 ± 2 °C for 15 min and filtered for a liquid extract. Dye solutions were prepared by adding 0.1% of polyethylene glycol 40 castor oil to the liquid extract to obtain a final concentration of 25 mg/L of total anthocyanins.

### Scanning electron microscope (SEM)

Hair samples (about 10 mm long) were stubbed onto sample holders, gold-coated and vacuumed prior to scanning by SEM (Hitachi S-3000, Japan).

### Atomic force microscopy (AFM)

The overlapping distances of the cuticle cells of hair samples, fixed onto sample holders, were randomly analyzed within a 5 μm x 5 μm area by the cantilever of the AFM with XEI Image Processing and Analysis program (Park System XE120, Korea) using contact mode with 400 N force onto the cantilever tip. Each sample was stored at 40% RH at 25±2 °C before use.

### Tensile properties

Hair shafts were soaked in water (pH 6.7), KOH solution (pH 12) or ash extract (pH 12) at 25 ± 2°C for 1 h, then blotted dry prior to measurement. The hair fiber was held vertically with a 3 cm distance between the tensile movable grips of the texture analyzer (TA.XT Plus, U.S.A.) which applied a strain rate of 0.3%·s^-1^ in tensile mode until hair breakage. Results were plotted between force (N) and elongation (%) which were calculated from the percentage of the elongated distance at each point divided by its initial distance between the grips. Each measurement was obtained from 8–10 individual samples.

### X-ray absorption spectroscopy (XAS)

A bundle of 40 treated hair shafts (about 2.5 cm long) were mounted onto the sample holder, fixed by a sheet of polypropylene-film and analyzed by XAS at the SUT-NANOTEC–SLRI XAS Beamline (BL5.2) Synchrotron Light Research Institute (Public Organization), Thailand, using a InSb (111) double-crystal monochromator for sulfur *K*-edge measurement in fluorescence mode by a Vortex ME4 four element silicon drift detector. The photon energy was calibrated at 2481.4 eV for FeSO_4_. Low-energy configuration was applied throughout all experiments.

### Energy dispersive spectrometry (EDS)

Six samples of eucalyptus ash (about 1–2 mg each) or ash extract (about 0.01 mL each) were stubbed onto sample holders, dried, gold-coated and vacuumed prior to scanning using a scanning electron microscope (Leo 1450VP, Hurley, U.K.) with an energy dispersive X-ray spectrometer (Technai G2 20, FEI, Thermo Fisher Scientific, Oregon, U.S.A.) for elemental analysis.

### Synchrotron-based Fourier transform infrared (SR-FTIR) microspectroscopy

The hair samples, embedded in paraplast, cooled at 4 °C for 12 h and microtome cross-sectioned (5 microns thick), were placed on Ag/SnO_2_-coated IR reflective low-e slides (Kevley Technologies, Chesterfield, OH, U.S.A.) for SR-FTIR microspectroscopy (Nicolet 6700, Thermo Scientific^™^, Madison, MI, U.S.A.) and confocal infrared microscopy (Continuum; Spectra Tech, Oak Ridge, TN, U.S.A.) at BL14A1, National Synchrotron Radiation Research Center (NSRRC, Hsinchu, Taiwan). The acquisition of 128-scanned FTIR spectra of each hair section at 4 cm^-1^ resolution in the spectral range of 4000–650 cm^-1^ and the beam size of synchrotron IR radiation were defined and mapped through observation by a confocal aperture and focused to 20 × 20 μm^2^ by a 32 × Cassegrain objective and lateral step size 10 × 10 μm^2^. IR absorption of carbon dioxide and water vapor was reduced by an automatic atmospheric suppression (OMNIC^™^ 9.2, 2012, Thermo-Fisher-Scientific, Waltham, MA, U.S.A.) and dry nitrogen purge.

Amide I band of peptide bond of protein, rich in spectral information of protein secondary structures, including alpha-helix, beta-sheet, turns and bends and random coil of protein, were monitored and overlapped with the subcomponent bands in spectral rane of 1750–1600 cm^-1^. The band center of each subcomponent badn in amid I band was analyzed using Peak Resolve of OMNIC^™^ and using Voigt as basis function for resolving each subcomponent band spanned in the amide I band. FTIR spectra for each sample was used to determine the secondary structure of proteins and area under the curve of each secondary protein was estimated for relative peak area (%). The mean relative peak areas of the subcomponent bands obtained from hair samples treated with ash extract were statistically compared with those of the relevant data.

### Adsorption of anthocyanins

Adsorption of anthocyanins by hair samples was determined from total anthocyanin content remaining after soaking 10 mg hair samples in 1 ml of the dye solution for 1 h at 25 ± 2°C. Total anthocyanin contents of the dye solution before and after hair soaking were analyzed and subtracted for total anthocyanins adsorbed and divided by weights of the hair samples. Total anthocyanin content was analyzed by pH differential method (AOAC2005.2) [29]. In brief, samples were diluted with KCl-HCl buffer (pH 1) or acetate buffer (pH4.5) for spectrophotometric analysis using a microplate absorbance reader (Sunrise, Switzerland) at 520 and 700 nm. Total anthocyanin content, as cyanidin-3-glucoside equivalents (C3GE), was calculated using Eq (1):

$$Total\;anthocyanins\;(\frac{mg\;C3GE}{L})=\frac{A\times MW\times 1000}{\varepsilon \times 1}$$ (1)

where A = (Abs_520nm_-Abs_700nm_) _pH1.0_ − (Abs_520nm_-Abs_700nm_) _pH4.5_, MW = molecular weight (449.2 g/mol for cyanidin-3-glucoside), DF = dilution factor, 1000 = factor for conversion from g to mg, ε = molar extinction coefficient (26900) in L mol^−1^ cm^−1^, and 1 = path length in cm.

The average total anthocyanins adsorbed by ash-treated hair were statistically compared with those by water-treated hair.

### Colorimetry

For determination of dyeing efficacy of the hair samples, LAB color space values corresponding to lightness/darkness (L*) and colors (red/green; a* and yellow/blue; b*) were obtained using a diode-array spectrophotometeric colorimeter [30] setting at a range of wavelengths between 400–700 nm, 0.1 nm precision and 8 degree. For each of the color determinations, hair samples were prepared in bundles of 40 hair shafts (> 2 cm long) before pretreatment and dyeing for 4 h, followed by rinsing with water and air drying. Values represent the average of 10 replicates for each treatment. Differences in lightness/darkness or L* (dL), red/green or a* (da) and yellow/blue or b* (db) of the treated hair samples were calculated from spectral data using water-treated hair as the reference. The overall color difference (DE*) of each treatment was calculated, using Eq (2):

$${DE}^{*}=\sqrt{dL^{2}+da^{2}+db^{2}}$$ (2)

The average L*, a* and b* of ash-treated hair were statistically compared with those by water-treated hair.

### Statistics

Linear regression was used to analyze for correlation between two variables. Validation was estimated by precision of the data. Student’s t-test was used to compare between means of parametric data while chi-square test for non-parametric data and the significant level was determined at 0.05.

## Results

**Fig 1:**
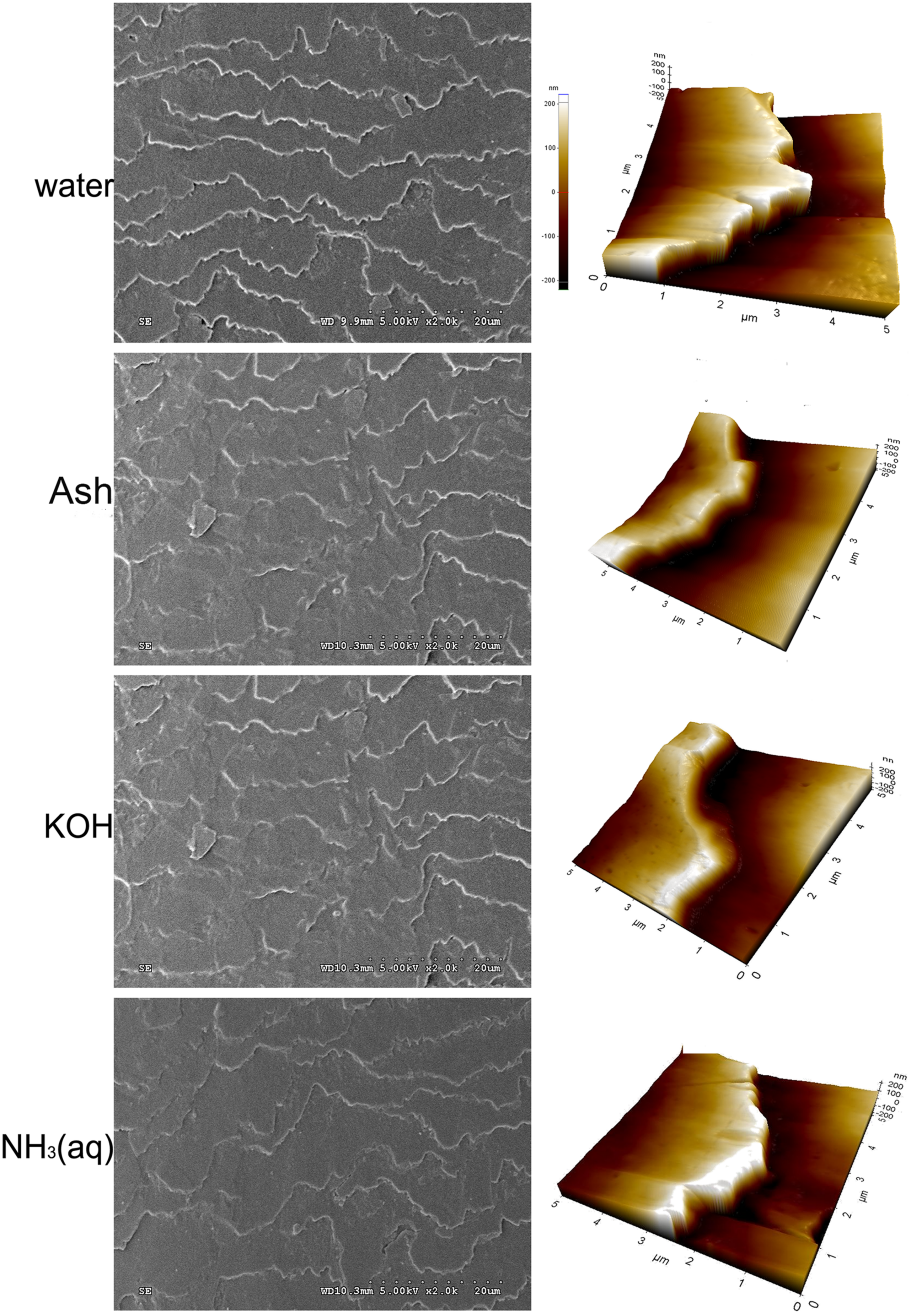
SEM and AFM images. Representative images from scanning electron microscopy (2000× magnification) (left column) and atomic force microscopy topography (contact mode, 400 N force on the cantilever tip in a 40 μm × 4 μm area randomly integrated for overlapping distances of 3 cuticle cells within 5 μm × 5μm) (right column) of hair samples treated with water (pH 6.7), ash extract (pH 12), KOH (pH 12) and ammonia solution (NH₃(aq), pH 12) at 55% RH and 25±2°C.

Fig 1 illustrates the surface morphology of grey hair samples treated with water, ash extract, KOH and NH₃(aq) solutions. SEM images of the hair show plate-like cuticle cells with well-defined ridges, between overlapping cuticle cells, perpendicular to the cuticle surface. The ridges of ash-treated, KOH-treated and NH_3_(aq)-treated hair were not as clearly defined as the control (water-treated hair), Fig 1 (left column). AFM 3-D images, Fig 1 (right column), the cuticle ridges of ash-treated, KOH-treated and NH_3_(aq)-treated hair appeared to be smoother than that of the control. The average ridge height of cuticles in water-treated hair (383.5 ± 23.6 nm) was slightly higher than those recorded for hair treated with either ash extract (354.5 ± 16.4 nm), KOH (378.0 ± 40.1nm) and NH₃(aq) (376.7 ± 18.3 nm), by AFM topographic analysis.

**Fig 2:**
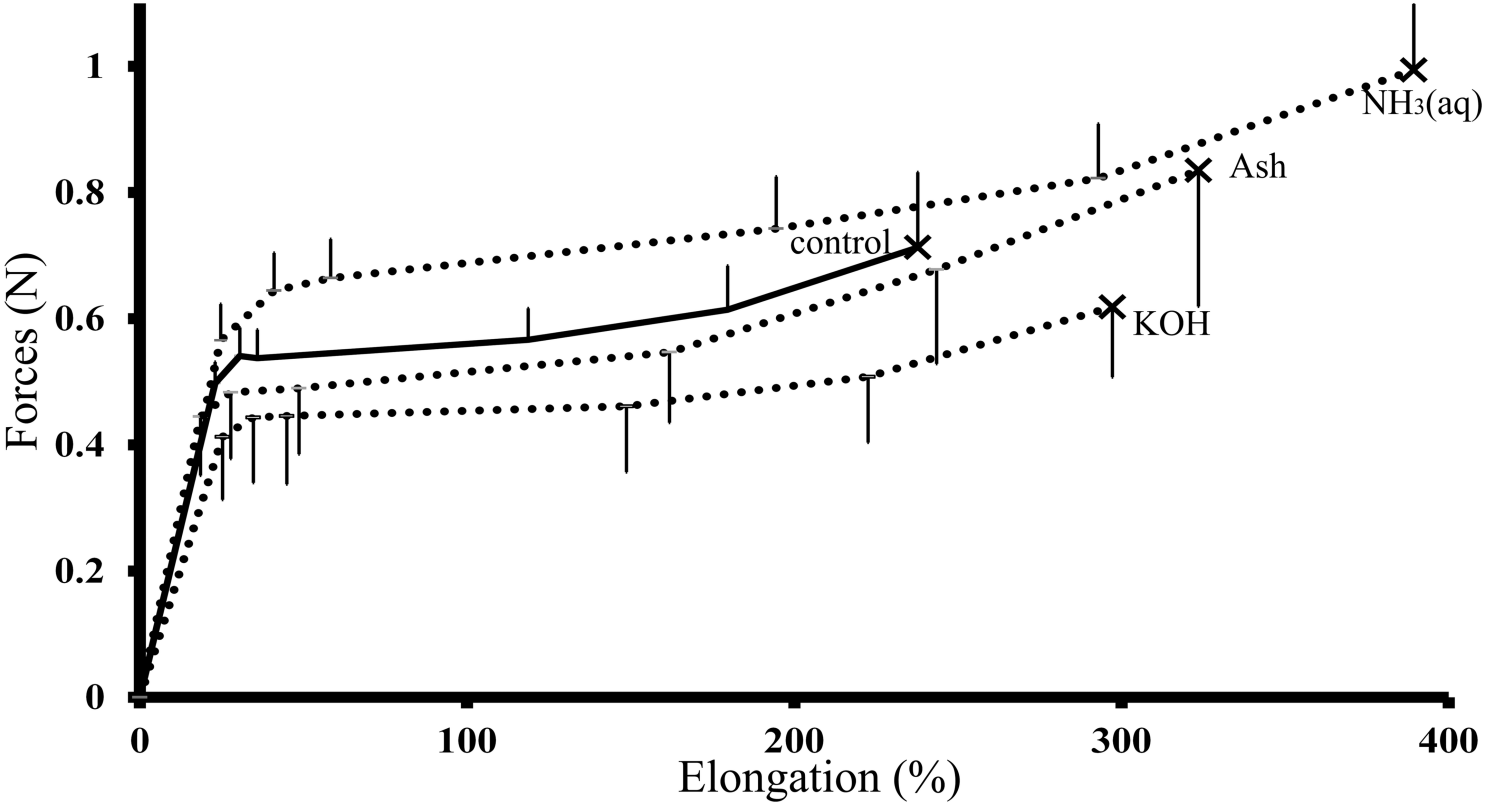
Tensile characteristics. Tensile characteristics of hair samples treated with water (pH 6.7) (control), KOH (pH 12), ash extract (pH 12) and ammonia solution (NH₃(aq), pH 12), at 25 ± 2°C; using texture analyzer (n = 8–10 each, 55 ± 3%RH and * = *p* < 0.05), error bars representing standard deviations.

Fig 2 compares the tensile characteristics of hair fibers treated with ash extract to those treated with KOH, NH₃(aq) and water (control). Ash extract and the other alkali treatments increased the maximum elongation distance of the hair fibers and the force exerted at maximum elongation distance was higher for NH₃(aq)-treated hair than the control. The force required to break the hair fibers, indicating tensile strength, was reduced in KOH treated hair but increased in NH₃(aq)- and ash-treated hair.

**Fig 3:**
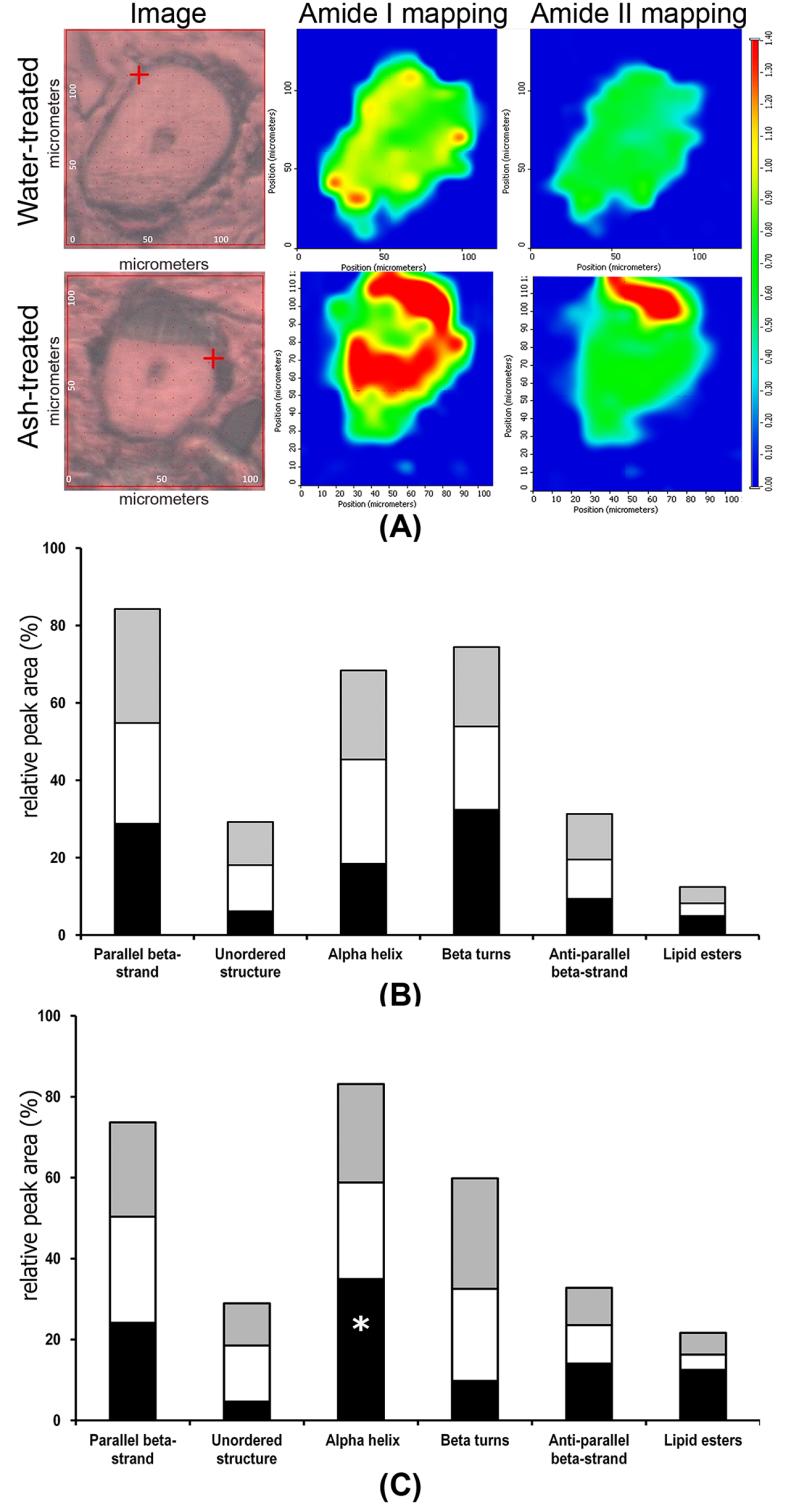
SR-FTIR micrographs. (A) Micrographs and FTIR spectral images of amide I and amide II bands, derived from 128 SR-FTIR spectra of cross-sectioned grey hair samples (5 μm thick) treated with water (control) and ash extract (ash-treated); (B) and (C) relative peak areas (%) of parallel beta-strand, unordered structure, alpha-helix, beta-turns, anti-parallel beta-strand and lipid esters distributed in the cuticle (black), cortex (white) and medulla (grey) regions deconvoluted from the SR-FTIR spectra of the hair samples treated with water and ash extract, respectively. All hair samples were treated for 1 h at 25±2°C, blotted-dried and stored at 55%RH until use. (the cuticle (marked ∪), the cortex (co) and the medulla (m), * *p* < 0.05, compared to the control).

SR-FTIR microspectroscopic analysis defined the cuticle, cortex and medulla layers in the greyscale cross-sectioned images (Fig 3A, left column), and shows increases in the intensity of amide I (1676 cm^−1^) and amide II (1550 cm^−1^) in hair shafts following treatment with ash extract (Fig 3A, right column). The uneven distribution of amide I and amide II in the cortex of ash-treated hair was observed in the associated spectral images that use color to illustrate spectral absorbance (Fig 3A). The SR-FTIR spectral intensities of amide bands for each sample region (the cuticle, cortex and medulla) were resolved into subcomponent bands assigned to lipid esters (1712–1715 cm^-1^) and secondary structures of proteins, which included the bands of 1693–1694 cm^-1^ and 1638–1641 cm^-1^ assigned to anti-parallel beta-strand and unordered structure, respectively, both bands of 1679–1682 cm^-1^ and 1627–1629 cm^-1^ assigned to parallel beta-strand, 1666 cm^-1^ assigned to beta-turns, 1650 cm^-1^ assigned to alpha-helix, 1612 cm^-1^ assigned to intermolecular beta-sheet (aggregation band) [19,31,32], and are presented in Fig 3B and 3C. Alpha-helices of ash-treated hair were significantly increased in the cuticle, compared to those in the control (p < 0.05). Ash-treated hair insignificantly increased relative peak areas of lipids (3000–2800 cm^-1^)/lipid esters (1712–1715 cm^-1^) in the cuticle layer (p > 0.05). An increase in relative peak areas of the anti-parallel beta-strand in the cortex and a decrease in those of the parallel beta-strand in the medulla of ash-treated hair were insignificant (p > 0.05).

**Fig 4:**
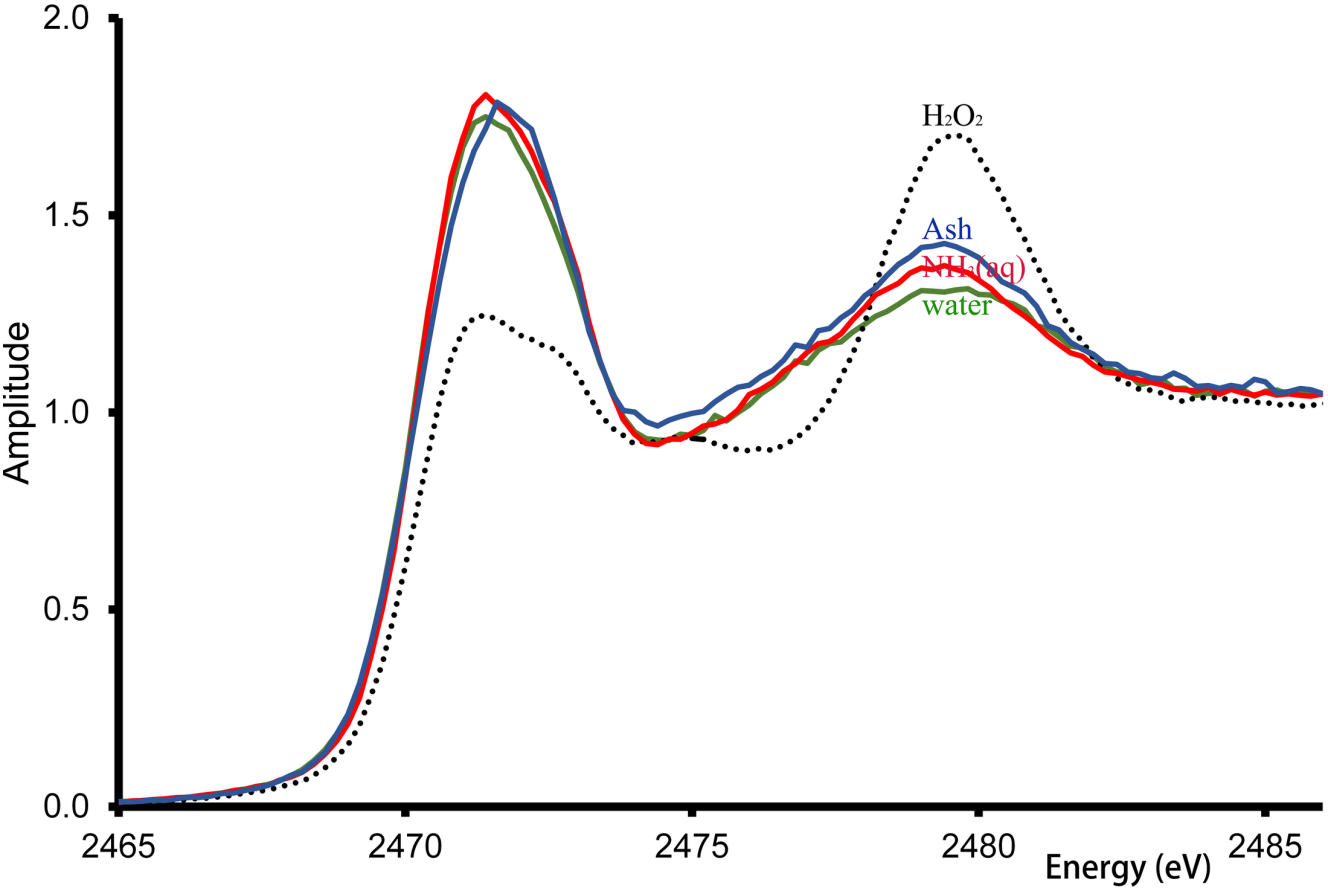
Sulfur K-edge XANE spectra. Sulfur *K*-edge XANES spectra recorded directly from the grey hair samples, 30-min treated with water, 30% ammonia solution (NH_3_(aq)), ash extract (Ash) and 3% hydrogen peroxide solution (H_2_O_2_) at 25 ± 1°C.

The sulfur *K*-edge XAS spectra, Fig 4, showed non-significant differences in ash-treated hair and the hair treated with NH₃(aq) and water between 2471.4 and 2479.4 eV (p > 0.05, all). The hair treated with H_2_O_2_, used as the positive control, exhibited spectral deviations from that of water-treated hair, i.e. an absorbance reduction at 2471.4 eV and an increased absorbance in the second absorbance peak combined with a small energy shift from 2479.4 to 2479.6 eV.

**Fig 5:**
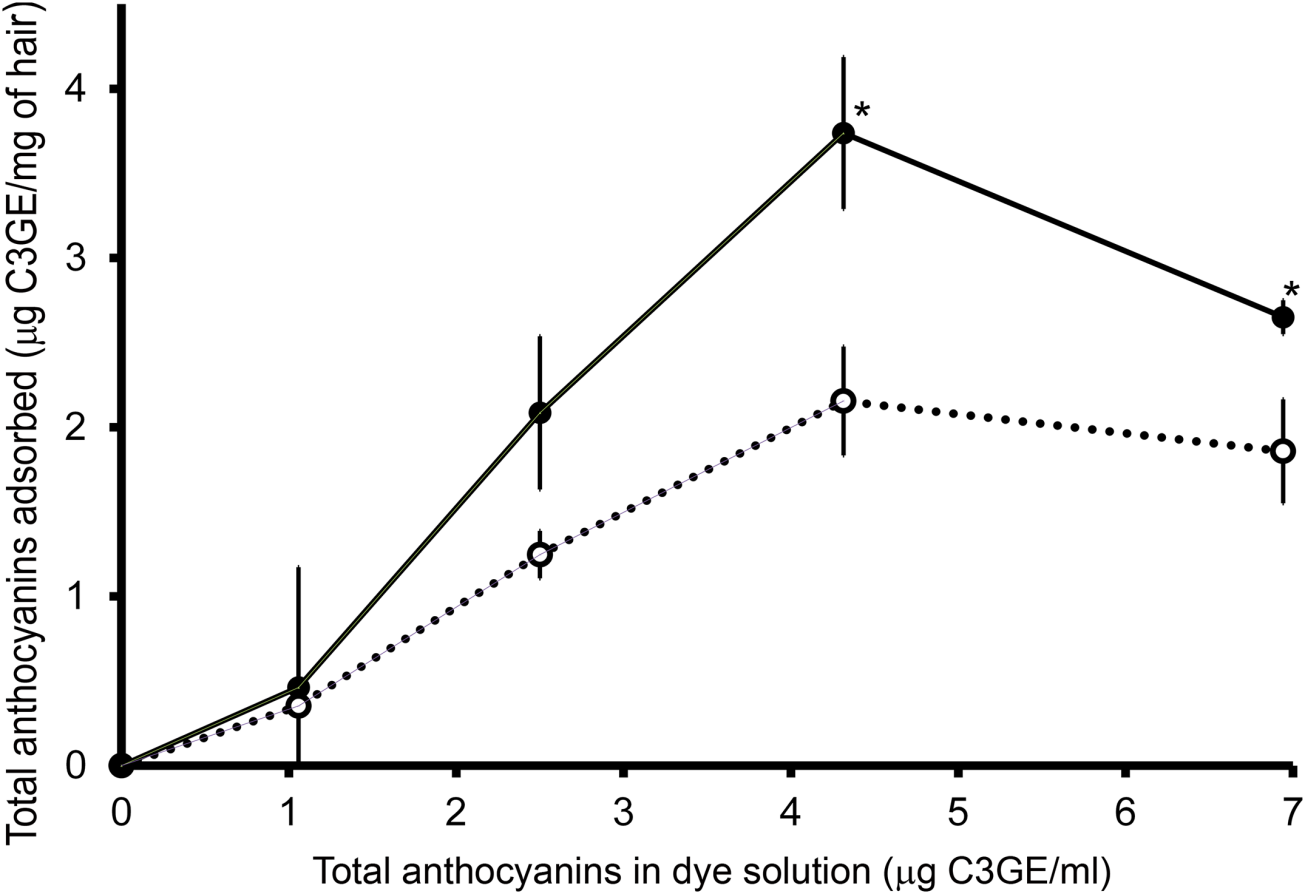
Anthocyanin adsorption. Total anthocyanin content (as C3GE) adsorbed by grey hair (μg/mg of hair) and concentration of total anthocyanin contents at initial (conc, μg/ml) compared ash-pretreated grey hair (**−●−**) and control (^**…**^**○**^**…**^), at 25 ± 2 °C for 1 h, n = 5 each and error bars = standard deviations and * = *p* < 0.05.

**Fig 6:**
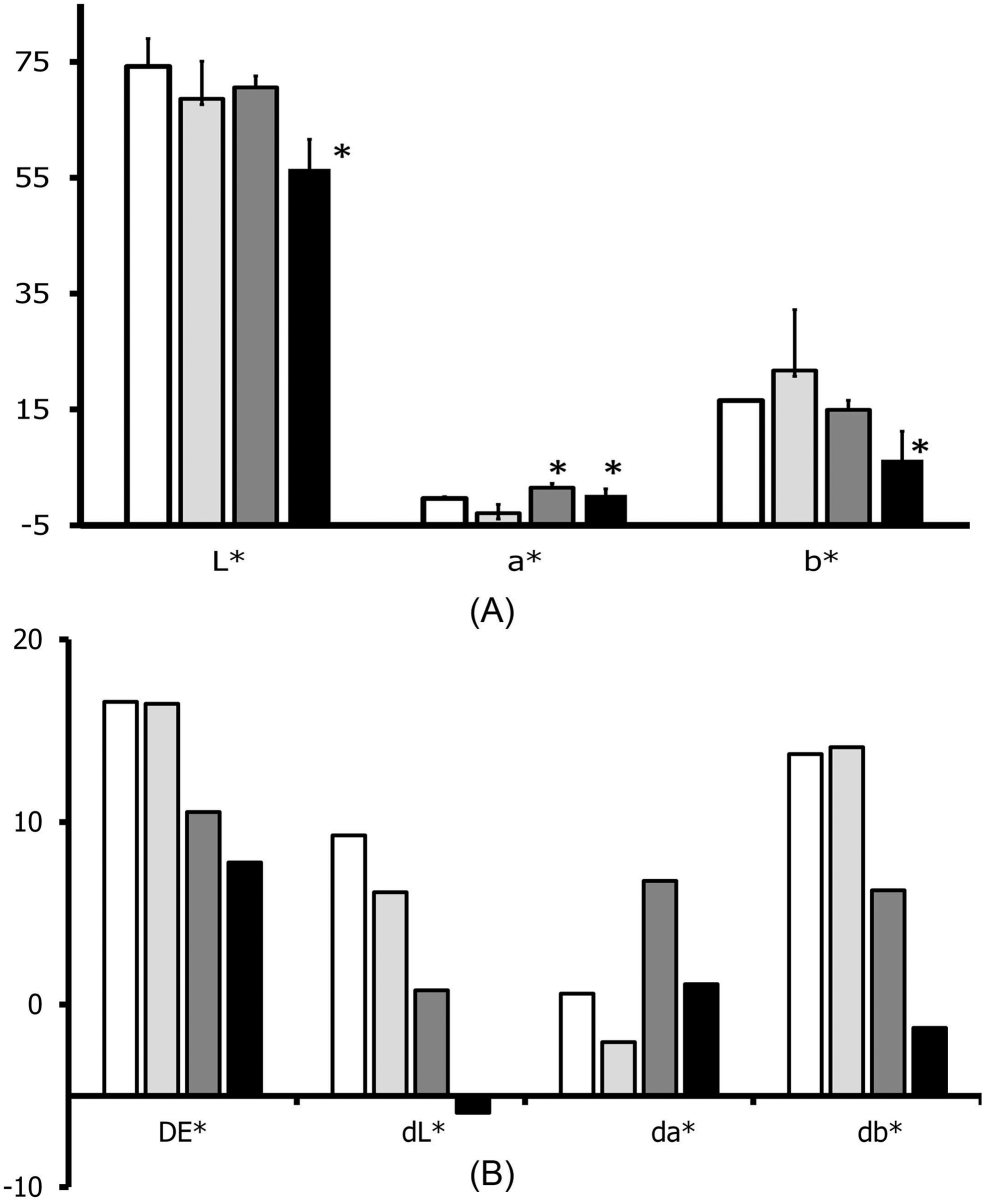
CIE color scale assessment. (A) Average CIE Lab colorimetric data of grey hair (across 50 shafts/measurement, n = 3) 1-h treated with water (clear columns), ash extract (light grey columns), dye solution (dark grey columns) and ash extract followed by dye solution (black columns), 1 h for each treatment and (B) overall color difference (DE*) of each treatment, calculated by Eq (2); L* = darkness(0)/lightness(100), a* = green(-)/red(+) and b* = blue(-)/yellow(+).

The amounts of total anthocyanins from the dye solution adsorbed by ash- and water-treated hair linearly increased with the concentrations of total anthocyanins up to 4.5 mg C3GE/ml (Fig 5). From the dye solution which contained 4.5 mg C3GE/ml, 2.1 and 3.8 μg C3GE/mg of hair were adsorbed by water- and ash-treated hair, respectively. At the same concentration, adsorption of total anthocyanins of 0.26 and 0.57 μg C3GE per mg of NH_3_(aq)- and KOH-treated hair, respectively, were observed. It was not possible to detect anthocyanins adsorbed by NH_3_(aq)- and KOH-treated hair at the other concentrations. Colorimetric analysis of the hair samples treated with either water, ash extract, dye solution or a combination of the ash extract followed by the dye solution (ash+dye) is shown in Fig 6. On the analysis of Lab color scale, hair treated with either dye alone or ash+dye showed a change in the total color difference (DE*) compared to water treated and ash treated hair (Fig 6). For hair treated with dye alone, this overall color difference was due to a reduction in lightness (L*, p< 0.05) and a small shift in color from green to red (a*, p <0.05). For ash+dye treated hair, the overall color difference was due to the large reduction in lightness (L*,p <0.05), the small shift in color from green to red (a*, p <0.05) and a large shift in color from yellow to blue (db*, p < 0.05, Fig 6).

## Discussion

Eucalyptus ash altered human grey hair, microscopically and morphologically in a fashion similar to NH_3_(aq) and KOH, at a pH of 12 and about 25 °C. Solutions of weak alkalis, e.g. NH_3_(aq), are applied in hair cosmetics to swell the cuticle cells and allow access of dye molecules into the cortex which was enveloped by the plate-like cuticle cells. Our results show that alkaline solutions reduced the ridge heights between overlapping cuticle cells by 20–30% consistent with previous studies that have shown reduction of the overlapping gaps at the cuticle ridges of the alkaline-treated hair [15]. The outer cuticle layer of the hair is composed of overlapping flattened cuticle cells adhered to each other by the cell membrane complex (CMC) [19,33]. These overlapping gaps observed by SEM as ‘ridges’ and by AFM as ‘cliffs’ are filled with the CMC, which is composed of lipids structurally organized to possess an adhesive-like property. The cuticle cells themselves are mainly filled with keratins. Thus, it is likely that the alkali solutions interacted with both the lipids of the CMC and the proteins of the cuticle cells, decreasing the overlapping gaps.

Tensile characterization is used as a physical measure of hair deformation, involving hair strength or damage [12–14]. Applying longitudinal pulling forces to a hair fiber interferes with its intercellular adherence in three different stages as defined by Hook’s law [14,16]. In the initial reversible stage, the fiber length increases (by an additional 30% of its original length) without permanent damage. This is followed by the second, irreversible, stage in which the fiber extends to approximately 70% of its original length and the final hair breakage stage usually occurs after 80% extension [14]. Hair strength is affected by water sorption, which causes hair shaft swelling, while excessive or repeated chemical treatments can accelerate hair breakage. In our study, treatment with alkalis (pH 12) appeared to interfere with some hair components and soften the hair shafts, increasing the length that a hair could stretch. Also, tensile strength, the minimum force required to irreversibly deform or break the ash- and NH₃(aq)-treated hair was increased. Mild deformations are known to reduce adhesiveness and cohesiveness within the microfilaments of the hair, resulting in increased length at breakage and increased tensile strengths [15]. Water hydrates the hydrophilic amino acids of non-keratin proteins to swell. The inconsistent alterations within the hair fibers after soaking the hair with water reduce the resistance to mechanical forces, affecting hair strength [13]. Water was, thus, used as the control in all of the tests conducted in this study. KOH, however, reduced the tensile strength of the hair due to its strong alkalinity. Eucalyptus ash acted as a mild alkaline treatment of the hair. During mild deformation, there are reversible transitions of beta-pleated sheets and alpha-helices of the hair proteins [33,34].

The high brightness of the synchrotron source improves resolution of the chemical images and allows sensitive and precise detection of minor changes and distinguish the hair fibers [17,19]. SR-FTIR microspectroscopy for monitoring the changes in lipids and proteins [30,31] and SR-XAS for determination of sulfur bonds [20] were, thus, used to determine chemical changes in the hair after treatment with the ash extract. The amino acid cystine forms disulfide bonds in hair fibers that act as a backbone and are a key contributor to the strength of the hair fibers [10,14]. Hair treatment with the ash extract altered the biochemical distribution of lipids (and/or lipid esters) and proteins (amide I and amide II) among the layers of the hair. The increases in lipids/lipid esters of the cuticle and the medulla of the hair following the ash treatment, suggest that the ash extract could penetrate into the hair shaft and re-distribute the lipid contents of the hair. Significant alterations of alpha-helices of hair proteins in the cuticle layer (p < 0.05) indicate some reversible structural changes of the secondary proteins. The structural arrangement of hair proteins plays a vital role in the strength of hair fibers; beta-strands generally interact with adjacent beta strands and the side chains of alpha-helix strands, which protects the core backbone structure [34,35]. The cortex and the medulla layers are generally responsible for the mechanical strength of the hair [10–13]. The cortex is composed of organized microfibrils made of alpha-helical keratin embedded and cross-linked with globular proteins in an amorphous matrix [34,35]. The medulla is important for hair strength in coarse hair [36], although there are more airspaces in the medulla than the cortex [37]. After treatment with the ash extract, the grey hair showed extended stretching with some alterations in lipid esters (cuticle), alpha-helices (cuticle) and anti-parallel beta-strands (cuticle), suggesting alkaline effects on alpha-beta transition of the hair proteins. Alpha-beta transitions of keratins occur as the hair was subjected to an applied tensile stress [14]. Also, altered alpha-beta transitions of keratins could alter tensile characteristics of the hair [38]. In our study, it appears that decreases in alpha-helices observed with ash-treated hair was responsible for the alteration of the tensile properties of the hair.

The XANE spectroscopy using synchrotron radiation used in this study can illustrate the types of chemical bonds in sulfur atoms of cystine or cysteine in non-destructive hair samples. Sulfur atoms form disulfide linkages between cystine in the hair proteins as well as thioester linkages with lipids [39]. Disulfide linkages play vital roles in hair strength, which manifests as tensile characteristics. The sulfur *K*-edge XAS indicated only very slight alteration of the grey hair treated with NH_3_(aq) or ash extract, at 2471 and 2479 eV, which refer to thiol (—SH) and sulfite (R—SO_3_) groups, respectively, with no evidence of oxidative damage [39–41]. H_2_O_2_-treated hair lowered the FT amplitude of the first peak and increased that of the second peak, presumably due to oxidation of cysteine sulfur atoms. Thus, the thioester and cystinyl groups of the hair may have been slightly altered, leading to the extended hair stretches, but there was no disruption of the disulfide-bond of cysteine observed, which is responsible for the principal mechanical strength of the hair.

The human hair has a complex morphological structure, the outer layer being covered by several layers of flattened dead cells, appeared as scales, and held together by a complex of adhesive materials. The outermost cuticle layer, which protects the hair from external environment and acts to restrict penetration of natural dye molecules, is the main obstacle for hair coloring by semi-permanent hair dyes [42]. Increased penetration of natural dyes in ash-treated hair could be due to the alkaline pH of ash extract affecting the isoelectric point of the hair proteins (the isoelectric point of keratin being 3.7 [43]) resulting in the hair surface becoming negatively charged. The negatively charged hair proteins could then induce ionization of anthocyanins contained in the applied dye solution resulting in flavylium cations [23] which would promote adsorption through attractive dipole interactions. Adsorption occurs as the anthocyanins from the dye solution attached to the surface of the hair. Molecular modelling techniques reveal that the diffusivity and penetration of cationic dye molecules into human hair fiber is better than non-ionic and anionic dyes [44]. An increase in hair adsorption of total anthocyanins in this study obtained by using a pH 6.5 dye solution (up to 4.5 μg C3GE/ml, 25 ± 2 °C) affirms the penetration of anthocyanins into the grey hair samples.

Color assessment of the hair affirmed an increase in anthocyanin adsorption as dye solution significantly reduced lightness of ash-treated hair at a higher extent than the control (p < 0.05). The reduction in lightness of the ash+dye-treated hair is in line with the shift from yellow to blue (db*) and green to red (da*). These blue and red shifts of the dyed hair matched to the effect of anthocyanins contained in the dye solution which was used to treat the ash-treated hair, confirming that the dyeing was due to anthocyanins. Thus, eucalyptus ash enhanced anthocyanin adsorption, resulting in hair darkening.

Aerobic combustion of fresh bark of a *Eucalyptus camaldulensis* × *Eucalyptus urophylla hybrid* (H4) produced about 4.5% of its ash, which was shown to elevate pH of water in a dose-dependent manner at 25±2°C, predominantly due to potassium contents. Its alkalinity is stronger than NH_3_(aq), but not as strong as KOH, possibly due to the interference of other substances. The SEM-EDS of the ash (S2A Fig) and its extract (S2B Fig) show small peaks of carbon indicating incomplete combustion. Analysis of the data derived the composition of the ash extract to be C, O, Na, Mg, Al, Si, S, Ca and Fe of 4.6, 48.4, 7.4, 1.0, 10.6, 24.8, 0.2, 2.2 and 0.8 as weight % and 7.5, 59.1, 6.3, 0.8, 7.7, 17.2, 0.1, 1.1 and 0.3 as atomic weights %, respectively. Eucalyptus ash is composed of C, O, Na, Mg, Al, Si, P, S, K, Ca and Fe of 6.4, 41.6, 1.2, 4.5, 6.1, 5.2, 1.9, 1.3, 0.5, 18.4 and 12.4 as weight % and 12.2, 56.6, 1.2, 4.0, 8.7, 1.3, 0.9, 0.3, 10.0 and 4.8 as atomic weights %, respectively. The data obtained from EDS analysis indicate that there was some trace carbon presented in the evaluation of the eucalyptus ash and the soluble elements from the ash were found in the ash extract. The eucalyptus ash was fine, soft, odorless, grey powder with average particle size of 0.8 μm, containing high proportions of heavy metals, potassium and calcium (S1 Table and S1 Fig), with a linear relationship between the logarithm of potassium equivalents and pH (r = 0.998) and a slope of 0.9 (S1 Fig). In comparison to KOH with a slope of 1 (completely ionized) and ammonia with a slope of 0.54 (partially ionized), eucalyptus ash was considered a moderate alkalinizing agent. The inorganic contents of the eucalyptus ash are in line with those of 11 eucalyptus ashes reported with different sodium contents which could be affected by age and species of the plants and should be further investigated.

This was the first attempt to elaborate the influence of pH elevation by wood ash on physicochemical changes of hair and hair proteins and the effects on anthocyanin adsorption. It may be applied for use as a source of natural enhancers for penetration of semi-permanent hair dye substances. There are several limitations of this study. The pH used to prepare alkaline treatment samples was 12 at room temperature. This was determined based on the pH of NH_3_(aq), generally used in hair pretreatment. Our images of grey hair samples which were solely obtained from local Asian donators were similar to previously described AFM and SEM images obtained from virgin Asian hair [14,34]. It might be interested to explore the effect of ashes on hair pigments, melanin, as well as other ethnicities which have been reported to have different characteristics. Using soaked and dried or unsoaked hair samples in the comparison of hair strength by AFM, Asian scalp hair has shown to be the highest strength when compared to Caucasian hair and African hair [14]. Other factors influencing penetration of dye molecules into the hair fibers and dye fastness which are vital for product development should also be further investigated. The ability of the human hair to stretch without damaging is compromised in various hair cosmetics, particularly those involving alkaline treatments.

Eucalyptus ash at pH 12 did not physically, mechanically nor biochemically deteriorate grey hair samples. To further develop its use in hair cosmetics, it is essential to consider investigations on safety and formulations. Polyethylene glycol 40 castor oil, a neutral surfactant used in cosmetic products, was selected for use to promote viscosity and solubilization of the dye solution. Other ingredients with influences on pH should be studied. While the hair-dyeing effect with a natural source of anthocyanins was demonstrated, further tests using isolated and/or purified anthocyanins, e.g. cyanidin-3-glucoside, cyanidin-3-glucopyranoside, delphinidin-3-glucoside or delphinidin-3-glucopyranoside, should be precisely evaluated to define hair dyeing effects of the alkaline-pretreatment. Also, there are other challenges in natural dye ingredients which are potentially used in hair dye products apart from the cationic anthocyanins. Further investigations are essential to determine the safe use of eucalyptus ash as a natural alkaline ingredient in hair cosmetics, particularly cross-reactivity with common hair dye ingredients [45]. Ash from other plant sources may also affect the human hair and could be further explored. Although anthocyanins are safe for human use, some people could be allergic to anthocyanins [23], the application of anthocyanins as hair dye is expected to be safer than chemical hair dye.

Ethnicity, distance along the hair shaft and size of the hair shaft can all affect surface structure, friction and adhesion of the hair cuticles [35]. To reduce variations and for ethical reasons, the hair samples used in this study were selected by the predetermined criteria, i.e. 10–30 cm from the scalp. The diameter of the hair shafts was also controlled as reduction in hair shaft diameter is one of the visible signs of aging hair [15]. Thus, the hair samples in this study represented average Asian virgin hair from the middle of the distance along the hair shaft. Finally, hair pigmentation is controlled by intrinsic factors including gene expression [46] and extrinsic factors, e.g. radiation, premature differentiation or activation of a senescence program [47]. Hair graying is considered to be caused by the follicle’s inability to maintain amelanotic melanocyte stem cells in the outer sheath of the human hair follicle [47]. Modern hair dyes are challenged by several issues including formulations which could potentiate dyeing of non-pigmented hair which individually occurs resulting in gray or white hair mixed with dark or black hair the same head [48]. Therefore, the effects of the ash on various hair types are essential to be further explored.

### Conclusions

Eucalyptus ash was shown to be a mild-to-potent alkalinizing agent in comparison to ammonia, which was selected for comparisons as one of the most commonly used pre-treatments in hair cosmetics. Ash extract (pH 12) promoted hair stretching with increased pulling forces required at hair breakage. Ash-treated grey hair was morphologically altered with flattening of the overlapping ridges of cuticle cells. The distribution of the secondary proteins of ash-treated hair was altered, mainly in the cortex layer, and showed alpha-beta transition. There were significant changes in distribution of lipids or lipid esters in the cuticle and medulla layers of ash-treated hair. There were slight deviations in the way sulfur interacts with its neighboring atoms of the ash-treated hair. Enhanced adsorption of total anthocyanins of ash-treated hair, and resulting in darkening the hair was obtained at a higher extent than the control. As a representative model of wood ashes, alkalinity of eucalyptus ash is shown to have some potential as a hair dye pretreatment for a natural extract from purple corn cobs without excessive damage.

## Supporting information

S1 Data File (XLSX)

S1 Fig Comparative alkalinity. Effect of eucalyptus ash (×) on pH of water (initial pH = 6.7), at 25 ± 1°C, plotted against logarithm of concentration of potassium equivalent (mM) in comparison to potassium hydroxide (KOH, □) and ammonia (NH_3_(aq), ○) solutions, equations and correlation coefficients (r) obtained by linear regression analysis; error bars = standard deviations (n = 10).
(TIF)

S2 Fig Energy dispersive spectrometry (EDS). Representative spectra for elemental analyses of (A) eucalyptus ash and (B) ash extract by EDS attached to a scanning electron microscope.
(TIF)

S1 Table Physicochemical characteristics of ash of *Eucalyptus camaldulensis* × *Eucalyptus urophylla* hybrid (H4). (DOCX)
