# Supplementary figures and images for: Eucalyptus ash alters secondary protein conformation of human grey hair and facilitates anthocyanin dyeing
Source: PLoS One. 2018 Jul 2;13(7):e0199696. doi: 10.1371/journal.pone.0199696 (PMC6028099; doi:10.1371/journal.pone.0199696)

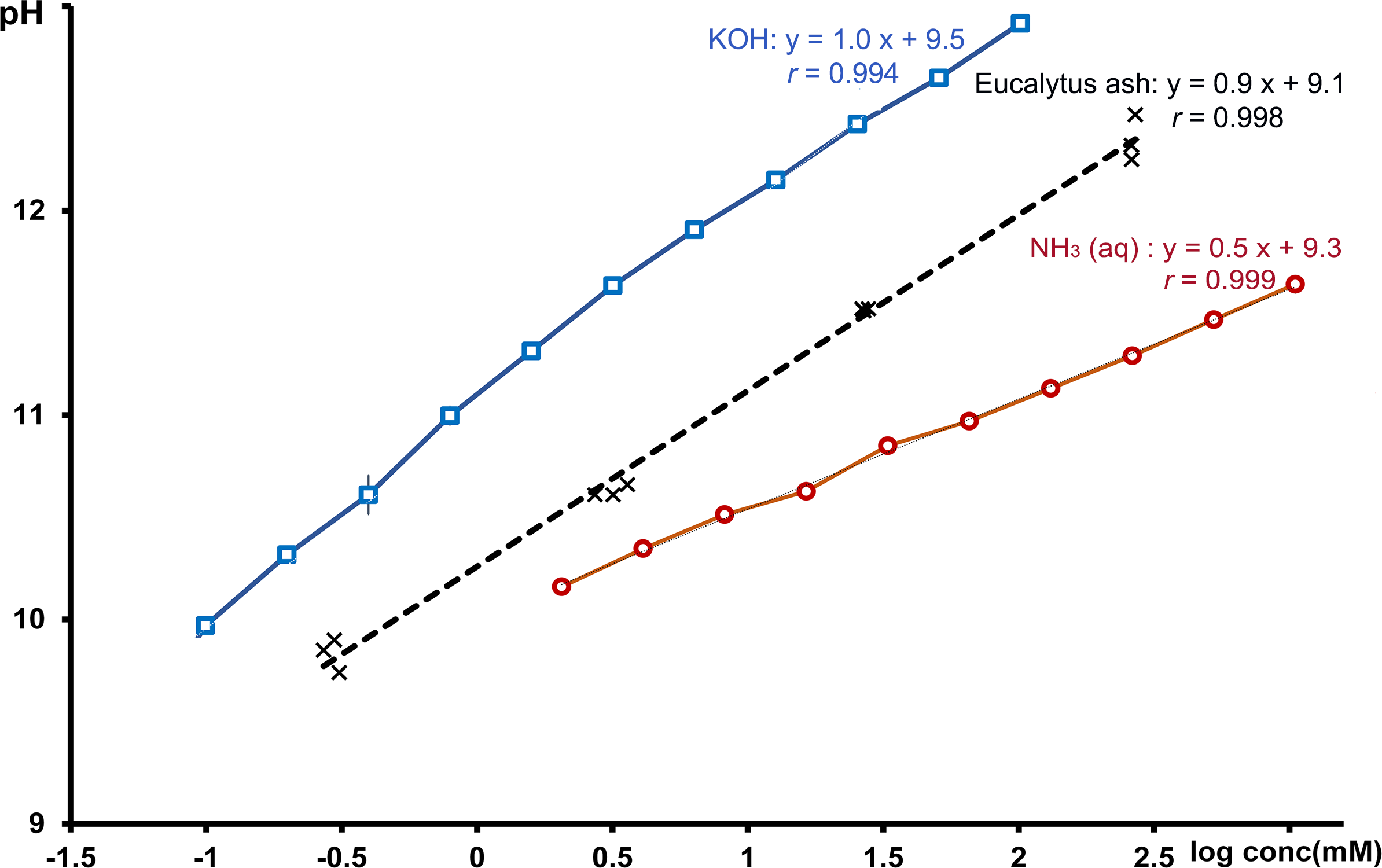

Supplement: S1 Fig — Effect of eucalyptus ash (×) on pH of water (initial pH = 6.7), at 25 ± 1°C, plotted against logarithm of concentration of potassium equivalent (mM) in comparison to potassium hydroxide (KOH, □) and ammonia (NH3(aq), ○) solutions, equations and correlation coefficients (r) obtained by linear regression analysis; error bars = standard deviations (n = 10). (TIF) [file pone.0199696.s002.tif]

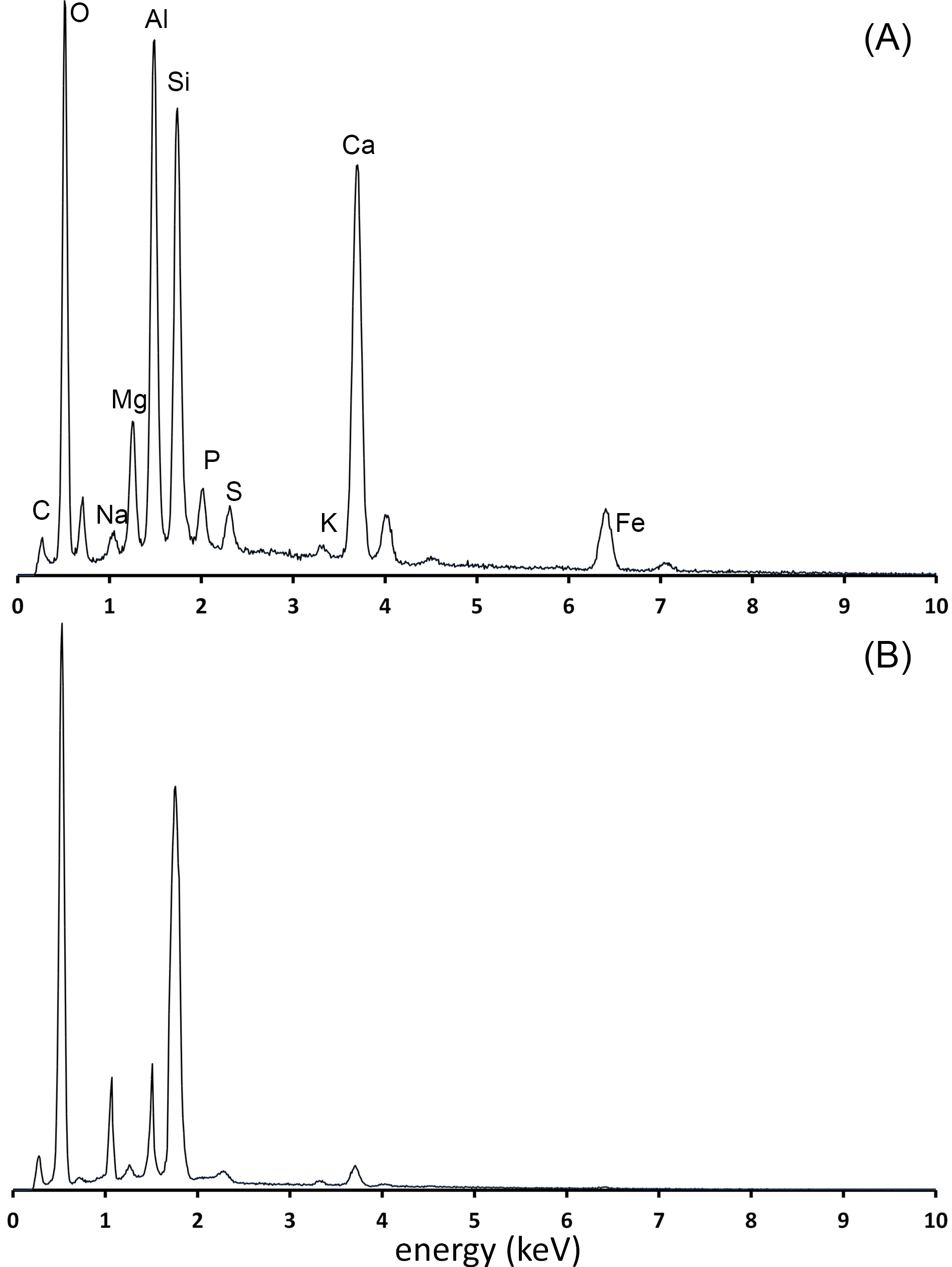

Supplement: S2 Fig — Representative spectra for elemental analyses of (A) eucalyptus ash and (B) ash extract by EDS attached to a scanning electron microscope. (TIF) [file pone.0199696.s003.tif]
