# Supplementary material for: Eucalyptus ash alters secondary protein conformation of human grey hair and facilitates anthocyanin dyeing
Source: PLoS One. 2018 Jul 2;13(7):e0199696. doi: 10.1371/journal.pone.0199696 (PMC6028099; doi:10.1371/journal.pone.0199696)
Supplement: S1 Table — (DOCX) [file pone.0199696.s004.docx]

**Table Supplementary 1 Physicochemical characteristics of ash** of *Eucalyptus camaldulensis* × *Eucalyptus urophylla* hybrid (H4).

| **Items** |  | **Eucalyptus ash** |
| --- | --- | --- |
| **Physical characteristics** |  |  |
| Particle size (μm, n = 60) |  | 0.79 ± 0.18 |
| Color |  | Grey |
| Appearance |  | Fine soft powder |
| Odor |  | Odorless |
| **Inorganic contents** (mg/g, n = 3) | | |
| Calcium (mg/g, n = 3) |  | 364.10 |
| Calcium oxide (mg/g, n = 3) |  | 509.74 |
| Iron (mg/g, n = 3) |  | 0.57 |
| Magnesium (mg/g, n = 3) |  | 15.49 |
| Manganese (mg/g, n = 3) |  | 9.52 |
| Phosphorus (mg/g, n = 3) |  | 11.38 |
| Potassium (mg/g, n = 3) |  | 73.59 |
| Sodium (mg/g, n = 3) |  | 2.53 |
| Zinc (mg/g, n = 3) |  | 0.07 |
| Arsenic (mg/kg, n = 3) |  | - |
| Cadmium (mg/kg, n = 3) |  | - |
| Lead (mg/kg, n = 3) |  | - |
| Mercury (mg/kg, n = 3) |  | - |
